# Trends in Income and Well-Being Inequality During the COVID-19 Pandemic in Japan

**DOI:** 10.1007/s11205-024-03478-6

**Published:** 2024-12-21

**Authors:** Kayoko Ishii, Isamu Yamamoto

**Affiliations:** 1 Faculty of Economics, Keio University, Tokyo, Japan; 2 Faculty of Business and Commerce, Keio University, Tokyo, Japan

**Keywords:** Inequality, Well-being, Income distribution, COVID-19, Japan, Causal mediation analysis

## Abstract

Although the COVID-19 pandemic could have caused both monetary and non-monetary distributional changes, existing studies have only investigated its immediate monetary impacts. This study examines the pandemic’s medium-term impacts on income and well-being inequality using individual longitudinal data from the Japan Household Panel Survey. Gini coefficients and income mobility before and after the pandemic are calculated to analyze income inequality. Various well-being measures such as mental health and life satisfaction are used to analyze well-being inequality. The findings reveal no increase in income inequality. Progressive income growth ensured stable inequality throughout the pandemic. Conversely, on average, well-being worsened, and well-being inequality increased. Furthermore, we find an association between income and well-being inequality. The random-effects and fixed-effects models indicate that the well-being of the high-income group tended to improve, whereas that of the low-income group tended to deteriorate after the outbreak of the pandemic. Additionally, the causal mediation analysis shows that the adoption of remote work served as a factor for the increase in the well-being of people in the high-income group. Remote work became disproportionately prevalent during the pandemic, especially among people in the higher income group. This group experienced various benefits of remote work, which contributed to an improvement in their well-being and an increase in well-being inequality.

## 1 Introduction

Owing to the enormous economic and social damage caused by the COVID-19 pandemic, several studies have investigated its distributional impact in many countries. Although some studies have shown an increase in income inequality even after considering regressive policy interventions (Angelov & Waldenström, 2023; Crossley et al., 2021), most indicate that income inequality after redistribution did not significantly increase (Almeida et al., 2021; Blundell et al., 2022; Clark et al., 2021; O’Donoghue et al., 2020; Stantcheva, 2022). As Clark et al. (2021) and Tanaka (2022) indicate, regressive policies using existing redistributive mechanisms and special intervention schemes fully offset the severe negative impact for lower-income households, suggesting that income inequality based on disposable income did not increase significantly, at least at the beginning of the pandemic. However, subsequent changes or the medium-term impacts of the pandemic are not known from the existing studies, which leads us to our first research question: whether the COVID-19 pandemic changed income inequality in the medium term.

In addition to income inequality, the importance of well-being inequality has been increasingly recognized in recent years. For instance, Veenhoven (2008) notes that individuals’ well-being and quality of life, not just their economic wealth, are important in assessing social equity, and this perspective adds a new dimension to inequality research. Several relevant studies have investigated the heterogeneous and immediate impact of the COVID-19 pandemic on well-being variables such as mental health, anxiety, general health, happiness, and satisfaction (Adams-Prassl et al., 2022; Banks & Xu, 2020; Nagasu et al., 2021; Yamamura & Tsutsui, 2022). For example, the pandemic had severe consequences for individuals with poor well-being before the pandemic, such as young people, women, and low-income households (Banks & Xu, 2020; Nagasu et al., 2021). This suggests a possibility that well-being inequalities increased during the pandemic. However, few studies have measured the medium-term distributional changes in these variables during the pandemic.^1^ This is relevant to our second research question: whether the COVID-19 pandemic changed non-monetary inequality such as subjective well-being in the medium term.

Considering the pandemic introduced “new normal” characterized by widespread but disproportionate expanding practices of remote work, changes in well-being inequality could be related to income inequality. Wealthier individuals, for example, may have had access to remote work, possibly enhancing their well-being. Bonacini et al. (2021) suggest that uneven remote work adoption during the pandemic risked exacerbating income inequality, benefiting high-income jobs more than low-income ones. Well-being inequality could further increase if remote work adoption continues disproportionately, with heterogeneous impacts on well-being. Despite varying findings, studies indicate that the benefit of remote work is heterogeneous depending on individual attributes (Esposito et al., 2024; Gueguen & Senik, 2023; Ishii et al., 2023; Senik et al., 2024), suggesting the need to explore the association between income and well-being inequality. This is connected to our third and main research question: whether changes in well-being inequality are associated with income inequality during and after the COVID-19 pandemic.

The possible association between income and well-being can be explained by the theoretical and empirical findings of Ishii et al. (2023). Using the random utility model in economics, they theorized that the exogenous shift to remote work due to the COVID-19 pandemic had a heterogeneous, rather than uniform, impact on workers. This heterogeneous impact was attributed to the amount of potential benefits of remote work, which depend on job characteristics, individual attributes, and institutional factors like insufficient devices and environment for online work activities. Before the pandemic, remote work was stigmatized as a form of shirking (Barrero et al., 2021; Kawaguchi & Motegi, 2021), leading to its limited adoption. However, as workers were compelled to adopt remote work during the first State of Emergency in April 2020, the stigma lessened as perceptions shifted. Consequently, those who benefited from remote work continued with it post-emergency, while others returned to the office. Ishii et al. (2023) supported these theoretical insights with empirical data from Japanese household panel survey, showing that workers with better IT skills, those engaged in abstract tasks, and those exposed to new technologies were more likely to benefit from remote work and continue it.

Based on Ishii et al.’s (2023) theoretical and empirical findings, we conjecture that the adoption of remote work was disproportionately observed for high-income groups because they tend to have higher IT skills, engage in more abstract tasks, and be more exposed to new technologies, as categorized by Ishii et al. (2023).^2^ Therefore, those with higher incomes likely experienced improved well-being during the pandemic because they could enjoy a better work arrangement with remote work. Thus, we hypothesize that an increase in well-being inequality during the pandemic is associated with income inequality through differences in the adoption of remote work.

Considering these research questions and the gaps in the existing literature, this study examines the medium-term changes in overall monetary (income) and non-monetary (subjective well-being) inequalities and their association during the COVID-19 pandemic in Japan. Representative longitudinal data from Japan, the collection of which started before the COVID-19 pandemic, allows us to analyze these impacts on income and well-being inequality considering pre-pandemic economic positions. As the well-being variables included in the data are self-reported, the use of a longitudinal survey that started before the pandemic is important to exclude retrospective biases. Unlike previous studies that have investigated immediate impacts on income distribution, we attempt to understand the medium-term impacts on inequality using data through 2022. This is based on concerns that the COVID-19 pandemic has changed the existing structure and trends of income and well-being inequality.

In the analysis, we first examine the changes in income inequality using three descriptive approaches: observing trends of the Gini coefficients, income fluctuations, and dynamic transitions or mobility among income groups. Second, we conduct a similar descriptive analysis for well-being inequality, observing changes in the mean and variation of well-being variables. Third, we examine an association between income and well-being inequality by focusing on remote work practice as potential factors that cause the association. After observing a descriptive relationship, we estimate random- and fixed-effects models for well-being variables such as mental health and satisfaction, in which we include pre-pandemic income quintile dummies as well as their cross-terms with the after-the-out-break dummy. We focus on the estimates of the coefficients of the cross-terms because they indicate how the pre-pandemic income level is associated with the changes in well-being after the outbreak. Then, we conduct causal mediation analysis with remote work practice as a mediator to decompose the total effects of income quintile dummies into natural direct and indirect effects, by which we confirm whether the increase in the well-being inequality across income quintiles was brought about by remote work practices during the pandemic.

The remainder of this paper is structured as follows. Section 2 reviews the literature, and Sect. 3 explains the analytical framework and data used in the analysis. Section 4 examines changes in income inequality and well-being inequality. Section 5 examines the association between income and well-being inequality. Finally, Sect. 6 presents the conclusions of this study.

## 2 Related Literature

This study is related to three strands of the literature. First, it contributes to the literature on the monetary distributional impact of the COVID-19 pandemic. Owing to the lack of timely available official data, early studies assessing the immediate impact of the pandemic and government interventions on inequality have used either microsimulation (Almeida et al., 2021; O’Donoghue et al., 2020; Palomino et al., 2020), rapidly collected survey data (Clark et al., 2021; Crossley et al., 2021; Gambau et al., 2022), or alternative data such as bank records (Aspachs et al., 2022) and high-frequency transaction data (Hacıoğlu-Hoke et al., 2021). These studies reveal that, although the pandemic had a severe impact on low-wage and low-income households, the policy response offset the regressive effect in the early stages of the pandemic. Studies using official statistics or representative data are limited, and most also focus on the immediate impact of the pandemic (e.g., Aina et al., 2023; Angelov & Waldenström, 2023). Bonacini et al. (2021) suggested the potential consequences of income inequality caused by remote work’s uneven adoption. However, little is known about the medium- and long-term impacts on income inequality.

Second, this study contributes to the literature on the pandemic’s impact on individual well-being. Several studies have investigated the heterogeneous impact of the COVID-19 pandemic on well-being in many countries (e.g., in the UK; Banks & Xu, 2020; Bu et al., 2020; Etheridge & Spantig, 2022; in the US; Adams-Prassl et al., 2022; in Germany; Huebener et al., 2021; Scmidtke et al., 2021; in Canada; Beland et al., 2022; and in Japan; Nagasu et al., 2021; Sudo, 2022; Yamamura & Tsutsui, 2022). However, most of these studies do not examine distributional changes in well-being during the pandemic.

In everyday life, diverse factors contribute to shaping well-being such as income, age, health, and social interaction (Dolan et al., 2008; Frey & Stutzer, 2002). During the pandemic, many factors such as restricted social interactions (Brodeur et al., 2021; Clark & Lepinteur, 2022), childcare burdens (Adams-Prassl et al., 2022; Senik et al., 2024), unemployment or financial worries (Adams-Prassl et al., 2022; Lee et al., 2021), and the threat of COVID-19 (Delhey et al., 2023) likely contributed to changing well-being. Remote work is also considered one such factor (Esposito et al., 2024; Gueguen & Senik, 2023; Ishii et al., 2023; Senik et al., 2024). There is a broad consensus across studies that remote work’s benefits are heterogeneous, depending on individual characteristics.

Third, this study contributes to the literature on well-being inequality. After Veenhoven’s pioneering study (1990), subsequent studies have clarified the negative association between well-being inequality and individual well-being within various group sizes (Delhey & Kohler, 2011; Dickinson & Morrison, 2022; Goff et al., 2018; Grimes et al., 2023; Okulicz-Kozaryn & Mazelis, 2017). However, little is known about the changes in well-being inequality during the COVID-19 pandemic, although Delhey et al. (2023) focused on the pandemic’s short-term impacts. Therefore, this study’s challenge is to identify the medium-term changes in overall income and subjective well-being inequalities and their associations during the COVID-19 pandemic in Japan.

## 3 Analytical Frameworks and Data

### 3.1 Analytical Framework

We first examine changes in income inequality and well-being inequality. Income inequality is measured by the Gini coefficient—commonly used for measuring income inequality—which ranges between 0 (perfect equality) and 1 (perfect inequality). Compared to other indices such as the mean log deviation and Teil index, the Gini coefficient is advantageous as it provides a clear, single summary of inequality across the entire income distribution and enables easy comparison between different populations or time periods. We also explored income fluctuations and mobility among income groups to identify whether income changes are occurring in a particular income group. Following Tanaka (2022) and Crossley et al. (2021), we analyzed income changes across income quintiles. We also decompose the change in inequality into the progressivity of income growth and re-ranking, following Jenkins and Van Kerm (2006).

There is no gold standard for measuring well-being inequality. Kalmijn and Veenhoven (2005) recommend standard deviation as the most suitable indicator. For maintaining comparability among four well-being variables, we use the coefficient of variation (CV), which is calculated by dividing the standard deviation by the mean. In the Appendix, other indices measuring well-being inequality, such as Jenkins’ Inequality Index (Jenkins, 2020) and percent maximum standard deviation (Delhey & Kohler, 2011), are also calculated and presented.

To analyze the association between income and well-being inequality, we first observe a descriptive relationship. Then, we estimate both random-effects and fixed-effects models for well-being variables, in which we include pre-pandemic income quintile dummies and their cross-terms with the after-the-outbreak dummy. We focus on the estimated coefficients of the cross-terms because they indicate how the pre-pandemic income level is associated with well-being changes post-outbreak. If the coefficients of the cross-terms of higher-income quintiles are significant, people in the higher-income group were likely to experience increased well-being more than those in the lower-income group during and after the pandemic. In such a case, the well-being inequality can be understood to have increased after the outbreak in association with pre-pandemic income inequality.

Next, to identify the factor that brings about the association between income and well-being inequality, we conduct a causal mediation analysis (Nguyen et al., 2020; VanderWeele, 2015). We set each well-being index as an outcome variable; the cross-term of pre-pandemic income quintile dummies and after-the-outbreak dummy (income quin-tiles) as an exposure variable; remote work practice as a mediator variable; and age, age squared, and dummy variables indicating sex, educational level, pre-pandemic income quintile, and year 2022 as control variables. Then, we decompose the total effect of the cross-terms of income quintile and after-the-outbreak dummy into natural direct and indirect effects. Specifically, we estimate the natural direct effect (NDE), which is the average direct effect of the treatment (income quintiles: *T*) on the outcome (well-being variables: *Y*) when the mediator (remote work: *M*) is held at 0 (remote work is not conducted): *Y*[*T*(1), *M*(0)] − *Y*[*T*(0), *M*(0)]. This indicates the effect of income quintiles on well-being through mechanisms other than remote work. Similarly, we estimated the natural indirect effect (NIE), which is the average indirect effect on the treated (income quintiles: *T*) through a mediator (remote work: *M*) on the outcome (well-being variables: *Y*): *Y*[*T*(1), *M*(1)] − *Y*[*T*(1), *M*(0)]. This indicates to what extent income quintiles affect well-being through remote work practice.

If only the natural indirect effect through remote work is significant, people in the higher-income group are likely better off because they practiced remote work more after the COVID-19 outbreak. That is, an association between income and well-being inequalities during the pandemic is because of the difference in the adoption of remote work among income quintiles. In such a case, as we conjectured, people in the higher-income group would have higher potential benefits from remote work, and therefore, their well-being would have improved after they conducted remote work.

### 3.2 Data and Variables

This study uses data from the Japan Household Panel Survey (JHPS). The JHPS is a representative longitudinal survey in Japan that follows the same individuals to track various changes such as working conditions, time use, health, well-being, and income. The JHPS began in February 2004. In the first year of the survey, approximately 4,000 adult men and women, along with their spouses, participated. The samples were selected using two-stage stratified random sampling methods to minimize selection bias. Since February 2004, surveys have been conducted every February. To compensate for sample attrition, new samples were added in 2007, 2009, 2012, and 2018.

To correct for the bias caused by the attrition, we employe sampling weights for all calculations that were created by an iterative proportional fitting or raking method to match the distributions of gender, age groups, working conditions, and number of people living together in each wave of the JHPS with those of the Japanese population. Population data were obtained from the Labor Force Survey (Ministry of Internal Affairs and Communications) for the same period. We fully matched the distributions of these variables to those from the Labor Force Survey using weights.

This study uses panel data from the 2020, 2021, and 2022 survey of the JHPS, and the sample used in this analysis was basically those who participated in the 2020 survey. The 2020 survey was conducted just before the outbreak of the pandemic; thus, it provides information from before the pandemic. The JHPS respondents were 20 years old and over at the time of sampling, and the sample sizes were 5,470 in 2020, 4,817 in 2021, and 4,517 in 2022, decreasing each year due to sample attrition. To capture overall trends in inequality for each year, we use information from all respondents unless information is missing and construct unbalanced panel data. The missing rate for each well-being variable is small (around 1%), but it is slightly higher in the question about income—about 10% in household annual gross income and 20% in household annual disposable income—since disposable income is difficult to ascertain in general. As shown by Ishii (2022), the use of the sampling weights brings the household income distribution in the JHPS data closer to that of the official statistics, thereby addressing the issue of missing data.

To analyze income inequality, we mainly use household annual gross income as its missing rate is much lower than household annual disposable income in the JHPS.^3^ Except for the analysis in Table 3, the household income is equivalized by dividing by the square root of the number of household members to reflect the differences in the needs of households of different sizes. As the survey asks about income over the last year, we refer to the year when the income was earned. The pre-pandemic income quintile is defined by the household gross income response in the JHPS 2020 survey.

To analyze well-being, we measure mental health, life satisfaction, job satisfaction, and health satisfaction. These four indices can capture a wide range of well-being. For mental health, we use the Kessler Psychological Distress Scale (K6), which comprises six questions measuring mental health, such as “Did you feel excessively nervous?” Responses are rated on a five-point Likert scale. The total score of the K6 ranges from 0 to 24 points, with higher scores indicating more severe mental problems. The three satisfaction scores about life, job, and health range from 0 to 10, with higher scores indicating higher levels of satisfaction. Table 1 presents question items for well-being variables.

For the causal mediation analysis, we use the remote work practice dummy variable as a mediator variable. This dummy variable takes the value of 1 if the respondent worked from home at least once in the fourth week of February in each year. Table 2 presents the descriptive statistics variables used in the estimation.

## 4 Income and Well-Being Inequality During the COVID-19 Pandemic

### 4.1 Trends in the Gini Coefficient

First, we examine the trends in income inequality before and during the COVID-19 pandemic using the Gini coefficient. We calculate the Gini coefficients using JHPS data and several official statistics to compare the results.

**Fig. 1:**
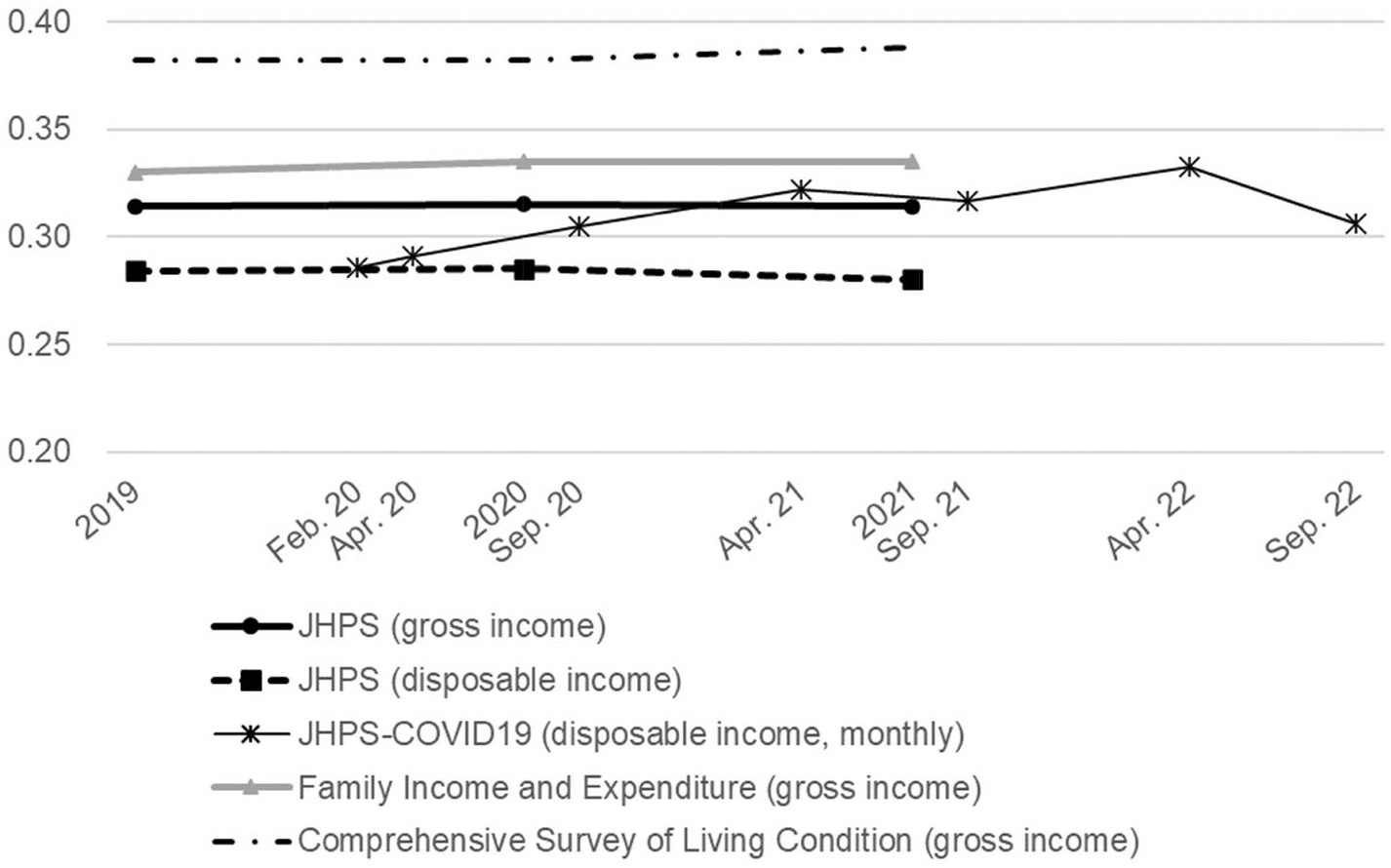
Gini coefficient before and during the pandemic. 1) Household income of the JHPS and the JHPS-COVID19 are equivalized by the square number of household members. 2) Gini coefficients are based on the household annual income, except for that by the JHPS-COVID19, which is based on monthly household income. *Source*: Authors’ calculation using the JHPS, the JHPS-COVID19, Family Income and Expenditure, and Comprehensive Survey of Living Condition

Figure 1 shows the Gini coefficients from 2019 to 2021 based on the JHPS. The Gini coefficients are mainly stable for both gross and disposable income before and two years after the pandemic outbreak. The Gini coefficient based on gross income is approximately 0.315. The coefficients based on disposable income are naturally smaller than those based on gross income, ranging from 0.280 to 0.285. Figure 1 also shows the Gini coefficients based on the equivalized monthly household gross income using data from the JHPS Special Survey for COVID-19 (JHPS-COVID19), which was conducted in April 2020 with the respondents of JHPS 2020 to capture the impact of the COVID-19 on households. The JHPS-COVID19 was conducted six times in spring and autumn from 2020 to 2022. The findings indicate a slight decrease in inequality in April 2020. Except for this, the Gini coefficients are mainly the same as those of the JHPS main survey.

Figure 1 also shows the Gini coefficients based on the government statistics. *Family Income and Expenditure* (Ministry of Internal Affairs and Communications) and the *Comprehensive Survey of Living Conditions* (Ministry of Health, Labour and Welfare) are used for government statistics. These two government statistics were the only surveys that examined income distribution during the COVID-19 pandemic in Japan. For these two Gini coefficients, income is defined as household annual gross income and is not equivalized by the number of household members.

As shown in Fig. 1, although the levels of Gini coefficient differ depending on the surveys, the changes in each Gini coefficient are small, within ± 2%. This corresponds to the changes from 0.314 to 0.314 in the levels of the Gini coefficient in the JHPS (gross income), those from 0.330 to 0.335 in *Family Income and Expenditure*, and those from 0.382 to 0.388 in the *Comprehensive Survey of Living Conditions*.

In summary, the results indicate that increases or decreases in the Gini coefficients were negligible during the pandemic. Thus, the COVID-19 pandemic did not impact income inequality in Japan.

### 4.2 Income Fluctuation During the COVID-19 Pandemic

While no overall increasing trend is observed in income inequality during the pandemic, significant changes may have occurred in income for a particular household or income group. Thus, following Tanaka (2022), we examine income fluctuations by income group during the pandemic.

Using aggregated data on *Family Income and Expenditure*, Tanaka (2022) calculates the changes in disposable income across income quintiles from 2019 to 2021 among the working population in Japan. Rows 1 and 2 in Table 3 (a) present the changes calculated using the same methods as Tanaka (2022), and Row 3 presents an extension to 2022. Current income (regular or replicable income that significantly affects households’ consumption behavior such as earnings, business income, asset income, and regular social security benefits) declined among the low- and middle-income groups from 2019 to 2020, whereas disposable income increased for all income groups. This is due to the increase in noncurrent income (irregular income, such as gifts) caused by the Special Cash Payment, which was government support that provided a fixed amount of 100,000 yen to all registered persons in 2020.

Current income continued to decrease from 2020 to 2021 in the low-income group, and the amount of income decrease since 2019 was the largest in the low-income group. Therefore, the negative impact of the pandemic was more pronounced in the low-income group. However, as shown in Fig. 1, this did not result in a large increase in overall income inequality. Based on the same statistics, the Gini coefficients among the working population were 0.227 in 2019, 0.232 in 2020, and 0.236 in 2021, indicating an increase of only 4% from 2019 to 2021.

The changes in both the current and disposable income of the middle-income group declined from 2021 to 2022, whereas those of the other income groups increased. The Gini coefficient among the working population was 0.220 in 2022, which was the same as that in 2019. These findings imply that income inequality increased slightly in the first two years of the pandemic; however, this increase was temporary, and the pandemic did not have a long-term impact on income inequality.

This tendency is confirmed by three additional aggregations. First, we conduct the same aggregation as Tanaka (2022) using the JHPS data in Table 3(b). In contrast to Table 3(a) and Tanaka’s (2022) study, this focuses on the total population (rather than the working population) and uses household annual gross income (not averaged monthly disposable income). Significant increases were observed in noncurrent income in all income groups from 2019 to 2020, which prevented a decline in total income except in the second and fifth quintiles. However, an income decrease was observed in almost all income groups from 2020 to 2021, except in the second quintile. Among all groups, the high-income group experienced the largest income decline since 2019. The Gini coefficients were 0.281 in 2019, 0.277 in 2020, and 0.276 in 2021, indicating a 2% decrease in the relative term. Therefore, the JHPS data confirm that the COVID-19 pandemic did not affect income inequality from 2019 to 2021.

**Fig. 2:**
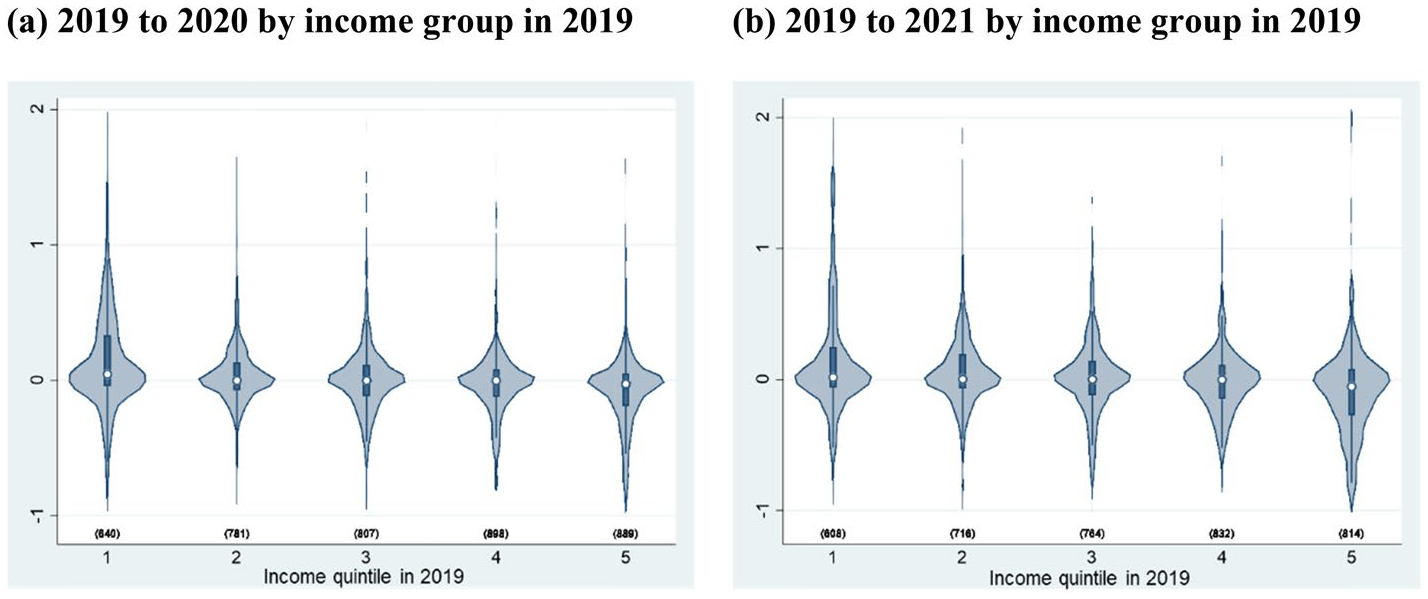
Violin plot of the percentage of income change by income group from 2019 to 2020 and 2019 to 2021 using the JHPS. 1) The definition of income in Fig. 2 is equivalized household annual gross income. 2) The income group is defined by the income in 2019. *Source*: Authors’ calculation using the JHPS

Second, taking advantage of the characteristics of the longitudinal data from the JHPS, we examine the dynamics of income fluctuations depending on the pre-pandemic income level, which is not confirmed by cross-sectional analysis. Figure 2 shows violin plots^4^ of the distribution of income changes from 2019 to 2020 and from 2019 to 2021 among the pre-pandemic income group based on Crossley et al. (2021).

As shown in Fig. 2(a), in all income groups, the modes and medians are located at almost zero, indicating that most households experienced no income changes. However, we also find that more households experienced income growth in the lowest quintile, whereas more households experienced income declines in the highest quintile from 2019 to 2020. By extending the observations from 2019 to 2021, as shown in Fig. 2(b), we find clearer tendencies. These findings indicate that, although the income of the low-income households at each point in time slightly declined during the pandemic, as shown in Table 3, households that were in the low-income group before the pandemic experienced more income growth during the pandemic. This provides evidence of stable income inequality through higher income mobility across income groups during the pandemic.

**Fig. 3:**
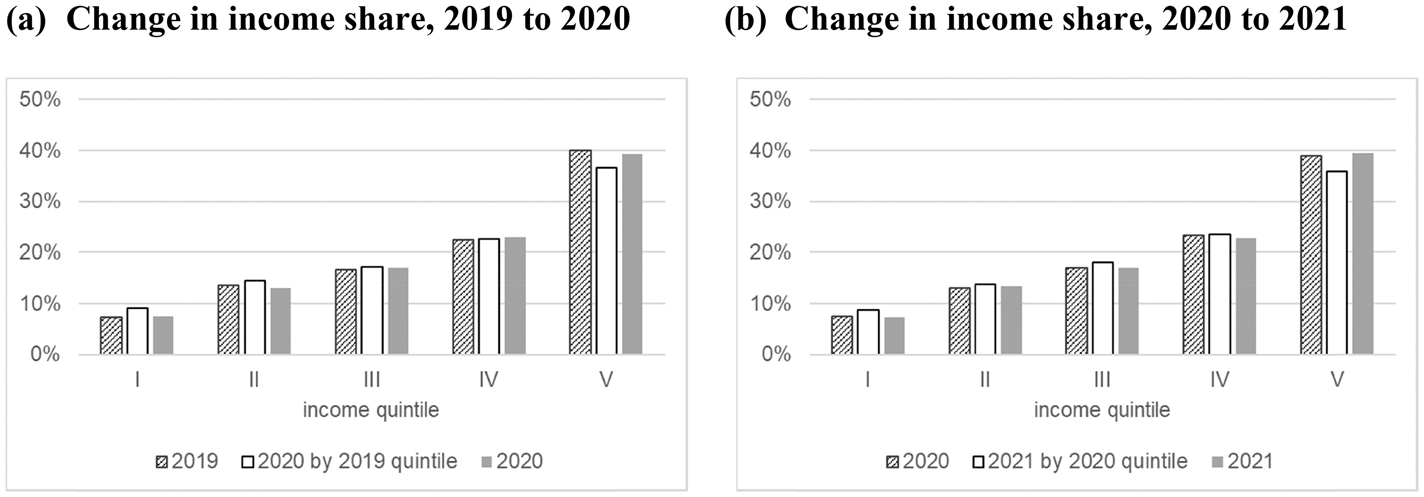
Changes in income share by income groups before and after COVID-19 using the JHPS. 1) The definition of income in Fig. 3 is equivalized household annual gross income. 2) These tables were calculated with two years of subsequent balanced panel data. *Source*: Authors’ calculation using the JHPS

Third, income growth and mobility are confirmed following the method presented by Jenkins and Van Kerm (2006). Jenkins and Van Kerm (2006) decomposed the change in inequality from time *t* to *t* + *1* into the progressivity of income growth and mobility in the form of re-ranking. Progressivity is expressed as the difference between the Lorenz curve at time *t* and the concentration curve of income at time *t* + *1* by income rank at time *t*, which indicates how much those experiencing poverty at time *t* grew their income share at time *t* + *1*. Re-ranking is expressed as the difference between the concentration curve of income at time *t* + *1* by income rank at time *t* and the Lorenz curve at time *t* + *1*. This is a comparison between the current income shares of those who were previously experiencing poverty and those who are currently experiencing poverty. Arranging this decomposition, we examine the changes in the income shares of each quintile directly, as shown in Fig. 3.

The income share is calculated in three ways: income share at time *t* by quintile at time *t*, income share at time *t* + *1* by quintile at time *t*, and income share at time *t* + *1* by quintile at time *t* + *1*. Comparing the first and second bars reveals how much the income share of households in each quintile in the first year changed in the next year. Likewise, by comparing the first and third bars, we find how much the income share of each quintile at each time point has changed. As shown in Fig. 3, in both periods, the households in the low- and middle-income groups in the first year increased their income share in the next year. Their income shares were greater than the income shares of those in the same position in the next year. Conversely, the households in the high-income group in the first year clearly experienced a decline in their income share in the next year. These changes in the income share provide additional evidence of mobility across income groups during the pandemic.

### 4.3 Trends in Well-Being Inequality

**Fig. 4:**
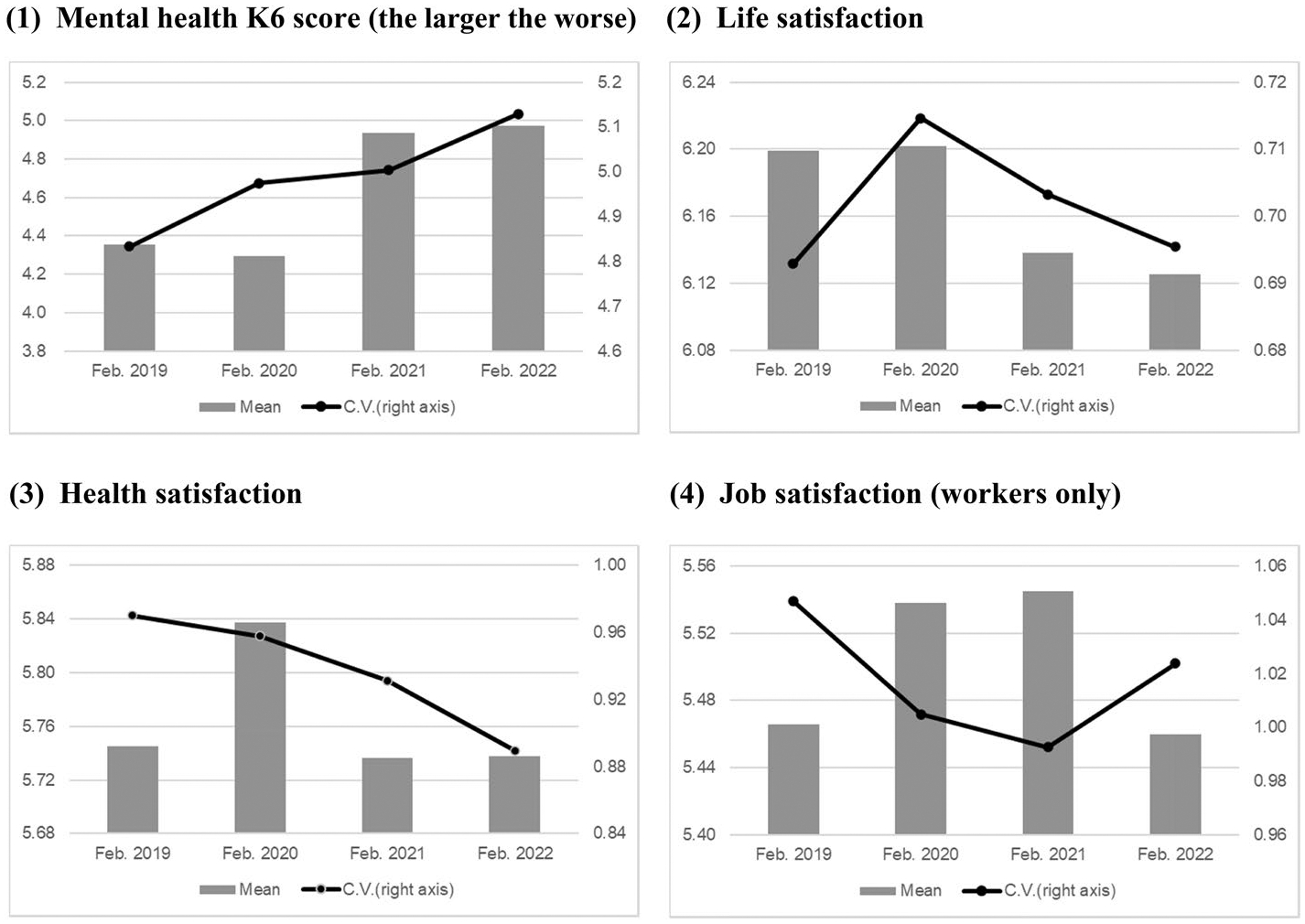
Means and coefficients of variation of well-being measures. *Source*: Authors’ calculation using the JHPS

To examine the change in inequality in terms of well-being, we first show the mean and coefficient of variation (CV) of each well-being variable before (February 2019 and 2020) and after (February 2021 and 2022) the pandemic.^5^ As shown in Fig. 4, the mean values of the K6 score, in which higher scores indicate worse mental health, increased in 2021 during the COVID-19 pandemic and stayed high in 2022. The CVs of the K6 exhibited an increasing trend, which was observed even before the pandemic. Therefore, inequality in mental health increased with average mental health deterioration during the pandemic.

Similar changes in the averages and variances of well-being are found for job satisfaction. Figure 4 shows a decrease in the mean values for both measures from 2021 to 2022, which indicates lower well-being, as well as an increase in the CVs, which indicates growing inequality during the pandemic.

As shown in Fig. 4, both the mean and CV of life and health satisfaction declined. This indicates that the average satisfaction with life and health decreased, although the inequality in those well-being measures shrank during the pandemic.

In summary, among the measures representing well-being, mental health (K6), life satisfaction, and health satisfaction worsened after the outbreak. The variance or inequality in mental health and job satisfaction increased. Thus, for some factors, differences between individuals in terms of well-being measures increased during the pandemic.

## 5 Association Between Well-Being and Income Inequality

### 5.1 Well-Being Inequality Associated with Income Inequality

**Fig. 5:**
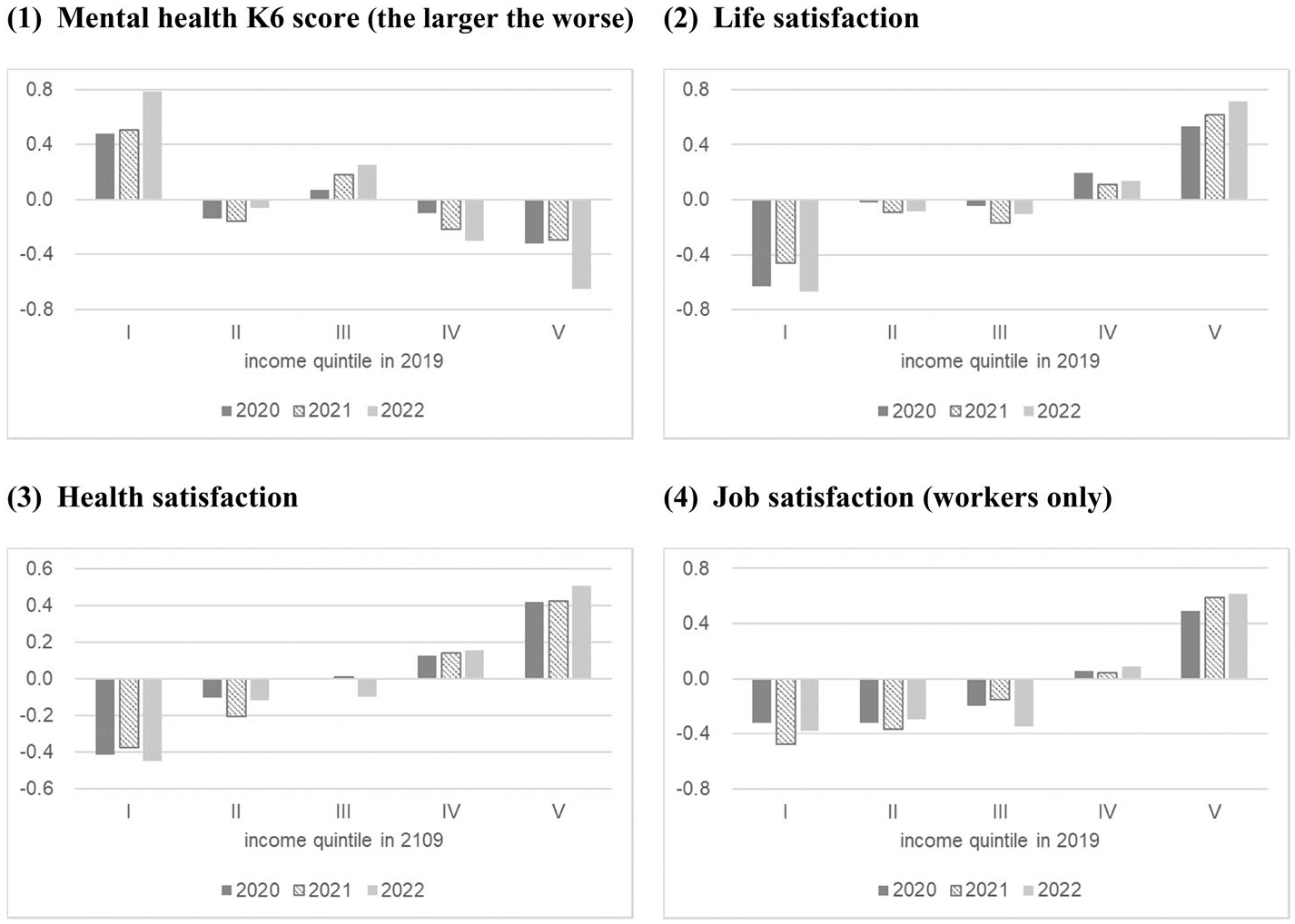
Mean deviations of each well-being from the total mean for each pre-pandemic income quintile group in February 2020, 2021, and 2022. Income quintile is calculated by the pre-pandemic income. *Source*: Authors’ calculation using the JHPS

To examine the association between well-being and income inequality, the mean deviations of each well-being measure from the total mean for each pre-pandemic income quintile group in February 2020, 2021, and 2022 are shown in Fig. 5.

Inequalities existed in pre-pandemic income levels in most well-being measures. Higher income level was associated with better well-being. Additionally, individuals in the high-income group before the pandemic experienced improvements in well-being, while those in the low-income group experienced a decline in well-being. For instance, we find a large decrease in the K6 score (better mental health) for the high-income group and a large increase (worse mental health) for the low-income group in 2022. Increases in overall inequality measured by the CV are not seen for life and health satisfaction in Fig. 4; however, an increase in the inequality associated with income inequality is observed in Fig. 5.

Table 4 shows the estimation results of the random-effects (RE) and fixed-effects (FE) models, which identify the association between income and well-being inequality by controlling for individual heterogeneity. We use data from 2020 and 2022 to examine changes from the pre-pandemic period to 2022. We use four well-being variables as dependent variables: mental health score (K6), life satisfaction, health satisfaction, and job satisfaction.^6^ As independent variables, we use dummy variables indicating the pre-pandemic income quintiles, the after-the-outbreak dummy (year 2022 dummy), and their cross-terms. We also control for age, age squared, sex, and educational level in the RE model. The coefficients of the dummy variables for the pre-pandemic income quintile indicate the inequality in each well-being variable in relation to the income level before the pandemic, as shown in Fig. 5. The coefficients of the cross-terms with the year 2022 dummy indicate whether well-being inequality in relation to income level changed during the pandemic.

Column [1] in Table 4 shows the results for mental health. The coefficients of the pre-pandemic income quintile are all significantly negative, and the magnitude of the coefficients increases with higher income quintiles, indicating better mental health for people in the high-income group. Thus, an association existed between mental health and income inequality before the pandemic. Furthermore, in columns [1] and [2], the cross-terms of the fifth quintile with the year 2022 dummy are significantly negative in the RE and FE models, which indicates that the mental health of people in the higher-income group improved more during the pandemic than did that of those in the low- and middle-income groups. Therefore, mental health inequality associated with income inequality increased during the pandemic.

Likewise, in the estimation results of the RE model for life satisfaction, health satisfaction, and job satisfaction in columns [3], [5] and [7], the coefficients of the pre-pandemic income quintile are all significantly positive, and the magnitude of the coefficients increases with higher income quintiles. These results indicate better satisfaction in terms of life, health, and jobs for people in the high-income group before the pandemic. The cross-terms of the fifth quintile with the year 2022 dummy are also significantly positive in columns [3], [5] and [6], suggesting that the life and health satisfaction of high-income individuals improved more during the pandemic compared to low-income individuals.

In summary, except for job satisfaction, the regression analyses indicate that the increase in well-being inequality was associated with income inequality during the pandemic, which is consistent with our hypothesis.

### 5.2 Causal Mediation Analysis for the Association Between Income and Well-Being Inequality

Table 5 shows the estimation results of the causal mediation analysis for identifying the factor that brings about the association between income and well-being inequality.^7^ For each well-being variable, the regression results of outcome and mediator models, as well as the decomposition results, are presented. For each regression, only the estimated coefficients of cross-terms of the quintile dummies with the year 2022 dummy are presented, representing the effect of the exposure variable (income quintile dummies after the outbreak) on well-being after controlling for the influence of the mediator (remote work dummy).

Regression results of the outcome model for mental health and life satisfaction in columns [1] and [5] of Table 5 show that the coefficients for the cross-terms of income quintiles with the year 2022 dummy are not significant. Note that those coefficients are significant in Table 4, where the remote work dummy is not controlled for, implying that people in the higher-income group were better after the outbreak of the pandemic in terms of mental health and life satisfaction, but such improvements were caused through the adoption of remote work during the pandemic. In fact, the coefficients of the remote work dummy are significant in columns [1] and [5], indicating that remote work adoption improved mental health and life satisfaction. Furthermore, the mediator model’s regression results, in which the remote work dummy is a dependent variable, show that the coefficients of the income quintile’s cross-terms and year 2022 dummy are significantly positive and larger for higher-income groups in all estimations. This implies that the people in the higher-income group tended to conduct remote work more after the outbreak.

This interpretation can be confirmed by the decomposition results of mental health and life satisfaction in Table 5, which indicate that the natural indirect effects (NIE) through remote work in columns [3] and [7] are significant for mental health and life satisfaction, while the natural direct effects (NDE) of income quintile in columns [4] and [8] are not significant. Specifically, the NIEs through remote work on mental health in column [3] are significantly negative and have larger absolute values in the higher-income group, indicating that people in higher-income groups are better off in terms of mental health because they conducted remote work more during the pandemic. Likewise, the NIEs through remote work on life satisfaction in column [7] are significantly positive and larger in higher-income group, indicating that people in higher-income groups are more satisfied in terms of life overall, because they were able to achieve more preferable remote working experiences.

For health satisfaction, decomposition results in columns [11] and [12] show the significant effects in both NIEs and NDEs, indicating that people in higher-income groups increased their health satisfaction during the pandemic partly because of remote work adoption, but other factors exist. For job satisfaction, no total effects of cross-terms of income quintiles and year 2022 dummy were observed in Table 4, but the decomposition results in columns [15] and [16] of Table 5 show that NIEs of remote work are significant and larger in the higher-income group while NDEs are not significant. Thus, people in the higher-income group tended to increase their job satisfaction if they conducted remote work.

In summary, inequality in well-being variables, such as mental health and life satisfaction, increased in relation to income level, and one of the drivers of this increase is identified as the availability of remote work. As such, the COVID-19 pandemic increased the association between well-being and income inequality through the inequality of workstyles and remote work.

## 6 Conclusion

This study investigated the distributional changes in income and subjective well-being over three years before and during the COVID-19 pandemic to explore whether the pandemic changed the existing structure and trends of inequality. Moreover, we examined the association between income and subjective well-being inequality by hypothesizing that the uneven adoption of remote work increased income inequality during the pandemic by disproportionately benefiting higher-income groups based on the theoretical and empirical findings by Ishii et al. (2023). The results revealed that income inequality did not increase and indicated that progressive income growth ensured the stability of income inequality throughout the pandemic. Well-being inequality increased during the pandemic and was associated with income inequality. That is, the well-being of the high-income group tended to improve, whereas that of the low-income group tended to deteriorate. The causal mediation analysis showed that the adoption of remote work served as a mediator for the increase in the well-being of people in the high-income group during the pandemic. Remote work became disproportionately prevalent during the pandemic among people in the higher income group, who originally experienced high potential benefits of remote work, which contributed to an improvement in their well-being and an increase in well-being inequality.

The study’s findings align with those of previous studies investigating the COVID-19 pandemic’s immediate impact on income inequality. Previous studies, such as Crossley et al. (2021), indicate negative impacts on income among low-income households in the pandemic’s early stages; however, overall inequality did not increase, partly owing to short-term policy intervention (Clark et al., 2021). Moreover, our findings demonstrate that the stability of income inequality continued for at least two years after the pandemic’s outbreak. According to Stantcheva (2022), income inequality in the medium and long term might increase owing to the regressive impacts of the pandemic; nonetheless, this was not observed in our study in Japan.

However, our analyses revealed an increase in inequality in non-monetary aspects, particularly subjective well-being. Inequality in non-monetary aspects has received less attention than monetary inequality in the literature on inequality. Similarly, Sudo (2022) measured the pandemic’s impact on subjective well-being in Japan, finding positive impacts for socially advantaged groups and negative impacts for socially disadvantaged groups, which increased well-being inequality. Moreover, other studies have reported that the increase in well-being inequality was associated with existing income inequality. For instance, Okulicz-Kozaryn and Mazelis (2017) demonstrated an increasing gap in happiness between income groups in the US.

Beginning with the pioneering work of Veenhoven (1990), a growing body of literature has investigated the development and distribution of non-monetary aspects, such as happiness, satisfaction, and health. Well-being is an outcome of life, and Veenhoven (2005) proposes that inequality can be measured using well-being rather than income. Our findings showed that the COVID-19 pandemic impacted non-monetary more than monetary aspects, suggesting the need to attend to distributional changes in subjective well-being, particularly when a society is hit by economic and non-economic shocks. Furthermore, our finding, that the increase in well-being inequality is associated with existing income inequality, suggests that, even if rising inequality is not observed on the surface, overall inequality, including that in non-monetary aspects such as happiness, satisfaction, and health, could be increasing. Therefore, more progressive distribution policies that effectively address both income and well-being inequality may be required.

We also found that the adoption of remote work is one factor contributing to shaping well-being. The pandemic introduced “a new normal” life with the expansion of remote work. This so-called “forced innovation,” as articulated by Bonacini et al. (2021), has become firmly established among high-income groups beyond the end of the pandemic, which is clearly confirmed in our mediation analysis. Our analysis also revealed that this disproportional adoption of remote work was a factor bringing about the association of the increase in well-being inequality with pre-pandemic income inequality. As confirmed by Ishii et al. (2023), remote work’s benefits can differ depending on individuals’ characteristics such as IT skills and job tasks: Those who have higher IT skills or engage in more abstract rather than routine tasks tend to have larger potential remote work benefits. Such people realized remote work’s actual benefits during the beginning of the pandemic, and then they continued to conduct remote work even when others with smaller potential remote work benefits returned to conventional office work. As a result, as we conjectured, such a disproportional adoption of remote work created an increase in well-being inequality. Thus, to reduce the well-being inequality associated with income inequality, increasing remote work’s potential benefit for people in lower-income groups is important. Enhancing human capital by investing in IT skill development, thereby enabling workers to engage in more abstract tasks, may serve as a strategic solution to amplify remote work’s benefits and further its promotion.

Although this study is one of the first to examine the medium-term impacts of the COVID-19 pandemic on income and well-being inequality in Japan, limitations exist. First, the sample size in our analysis is not necessarily large. Although we use representative longitudinal data from JHPS and apply the sampling weight to recover the population, the sample size is at most approximately 5,000, which is smaller than that of the surveys used by previous studies in other countries. Second, although we examined medium-term distributional changes with the data until 2021, the pandemic was such a devastating shock that it may have long-term effects on income and well-being. Future studies should investigate this further.

## Figures and Tables

**Table 1:** JHPS questions for well-being variables

(a) Kessler psychological distress scale (K6) for mental health index					
Q. The following questions ask about how you have been feeling during the past 30 days. For each question, please circle the number that best describes how often you had this feeling					
During the past 30 days, about how often did you feel …	All of the time	Most of the time	Some of the time	A little of the time	None of the time
a. …nervous?	1	2	3	4	5
b. …hopeless?	1	2	3	4	5
c. …restless or fidgety?	1	2	3	4	5
d. …so depressed that nothing could cheer you up?	1	2	3	4	5
e. …that everything was an effort?	1	2	3	4	5
f. …worthless?	1	2	3	4	5

(b) Satisfaction scores											
Q. Please provide answers as to how you feel about the present situation regarding the following, on a scale of 1 to 10, with 0 “not at all satisfied,” 5 is “neither satisfied nor dissatisfied,” and 10 is “fully satisfied” (circle one)											
	Dissatisfied ⇐										⇒Satisfied
Life overall	0	1	2	3	4	5	6	7	8	9	10
Your health	0	1	2	3	4	5	6	7	8	9	10
Your job	0	1	2	3	4	5	6	7	8	9	10

1) The Kessler Psychological Distress Scale (K6) is referred to from Kessler et al. (2002)

2) To calculate the K6 score, we first reverse the scores of each item so that a score of 1 becomes 4 and a score of 5 becomes 0, and then sum the scores for each of the six items to generate total score from 0 to 24. Higher scores indicate greater psychological distress

*Source*: The JHPS questionnaire

**Table 2:** Descriptive Statistics of the pooled data of JHPS from 2020 to 2022

	mean	S.D.	observation
*Income*			
Household gross income (10,000 yen)	669.9	492.3	13,222
Household disposable income (10,000 yen)	502.5	314.7	11,796
*Well-being variables*			
Mental health K6 score [0–24]	4.5	4.8	14,621
Life satisfaction [0–10]	6.2	2.1	14,707
Health satisfaction [0–10]	5.8	2.3	14,715
Job satisfaction (workers only) [0–10]	5.7	2.3	10,152
Working from work at least once a week	7.4%	0.3	13,707
(before the outbreak)	0.7%	0.1	
(after the outbreak)	11.7%	0.3	
*Individual characteristics*			
Age	55.3	16.0	14,803
Male	48%	0.5	14,803
*Educational level*			
High school graduate	48%	0.5	14,630
Junior college graduate	22%	0.4	14,630
University graduate or above	30%	0.5	14,630

This table refers to our estimation sample from the JHPS 2020, 2021, and 2022

*Source:* Authors’ calculation using the JHPS

**Table 3:** Changes in household disposable income by income group before and during the pandemic

(a) Working population (monthly income)		I	II	III	IV	V
2019–2020	Current income	− 1.40	− 7.10	− 7.78	10.66	10.71
	Non-current income	9.71	11.79	16.41	19.66	24.45
	Nonconsumption expenditures	− 0.27	1.08	− 1.23	5.89	6.58
	Disposable income	8.58	3.62	9.86	24.43	28.58
2020–2021	Current income	− 9.68	8.85	13.68	− 6.68	19.71
	Non-current income	− 6.85	− 9.06	− 14.71	− 14.71	− 17.47
	Nonconsumption expenditures	− 2.59	− 2.00	− 0.92	− 4.20	2.63
	Disposable income	− 13.93	1.18	− 0.11	− 17.20	− 0.39
2021–2022	Current income	22.36	9.75	− 2.13	20.66	15.34
	Non-current income	0.27	− 0.84	0.10	− 2.07	− 0.42
	Nonconsumption expenditures	4.33	3.47	1.58	5.50	3.26
	Disposable income	18.30	5.45	− 3.61	13.09	11.66
(b) Total population (annual income)		I	II	III	IV	V
2019–2020	Current income	− 18.5	− 159.7	− 11.1	107.6	− 271.2
	Non-current income	95.1	127.1	147.5	137.3	168.3
	Totalized household income	83.1	− 36.1	141.8	244.2	− 106.5
2020–2021	Current income	− 98.7	167.8	− 19.9	− 130.7	− 237.0
	Non-current income	− 66.2	− 70.5	− 105.4	− 99.4	− 68.0
	Totalized household income	− 172.0	92.9	− 111.9	− 228.0	− 298.8

1) The definition of income in Table 3(a) is averaged monthly household income, and the data source is Family income and expenditure

2) The definition of income in Table 3(b) is household annual gross income, and the data source is the JHPS

3) Current income in Table 3(a) is regular or replicable income that significantly affects households’ consumption behavior such as earning, business income, asset income, and regularly receiving social security benefit. Non-current income is irregular income such as donation and includes the Special Cash Payment

4) Current income in Table 3(b) includes earning, business income, asset income, interest, remittances, pension, and other regularly received social security benefits. Non-current income is that other than current income and includes the Special Cash Payment

*Source:* Authors’ calculation using the JHPS and Family Income and Expenditure, following Tanaka. (2022) Table 2

**Table 4:** Estimation results for the association between income and well-being inequality by controlling for individual heterogeneity

	Mental health K6 score (the larger the worse)		Life satisfaction			Health satisfaction		Job satisfaction	
	[1]	[2]	[3]		[4]	[5]	[6]	[7]	[8]
Pre-pandemic income quintile (ref: I & II)									
III	− 0.413**		0.387***			0.265***		0.210*	
	(0.188)		(0.0816)			(0.0927)		(0.117)	
IV	− 0.740***		0.652***			0.339***		0.495***	
	(0.183)		(0.0796)			(0.0904)		(0.110)	
V	− 0.884***		0.952***			0.648***		0.855***	
	(0.187)		(0.0813)			(0.0924)		(0.111)	
Cross-term of pre-pandemic income quintile and year 2022 dummy									
III * Y2022	− 0.0429	− 0.104		− 0.0601	− 0.0634	− 0.0398	− 0.0630	− 0.122	− 0.120
	(0.186)	(0.191)		(0.0740)	(0.0758)	(0.0842)	(0.0862)	(0.122)	(0.127)
IV * Y2022	− 0.260	− 0.276		− 0.0162	− 0.0556	0.145*	0.107	− 0.0105	− 0.0637
	(0.179)	(0.184)		(0.0713)	(0.0730)	(0.0810)	(0.0830)	(0.114)	(0.119)
V * Y2022	− 0.333*	− 0.339*		0.140*	0.112	0.218***	0.203**	− 0.00618	− 0.0165
	(0.181)	(0.186)		(0.0719)	(0.0737)	(0.0818)	(0.0838)	(0.114)	(0.120)
Year 2022 dummy	0.856***	0.755***		− 0.123***	− 0.0837*	− 0.193***	− 0.177***	− 0.00878	0.0379
	(0.111)	(0.114)		(0.0444)	(0.0454)	(0.0504)	(0.0516)	(0.0810)	(0.0855)
Individual effects	RE	FE		RE	FE	RE	FE	RE	FE
Observations	8,314	8,314		8,343	8,343	8,347	8,347	5,781	5,781
Number of IDs	4,801	4,801		4,799	4,799	4,802	4,802	3,496	3,496

1) ***, **, and * indicate statistical significance at the 1%, 5%, and 10% levels, respectively

2) RE indicates random-effects model, and FE indicates fixed-effects model

3) Age, age squared, sex and educational level are controlled in RE models

4) The sample is restricted to workers only for columns [7] and [8]

*Source*: Authors’ calculation using the JHPS

**Table 5:** Estimation results of causal mediation analysis for the association between income and well-being inequality

	Mental health K6 score (the larger the worse)				Life satisfaction				Health satisfaction				Job satisfaction			
	Regression results		Decomposition results		Regression results		Decomposition results		Regression results		Decomposition results		Regression results		Decomposition results	
	Outcome model (well-being)	Mediator model (Remote work)	Natural indirect effect (NIE) through remote work	Natural direct effect (NDE) of income quintile	Outcome model (well-being)	Mediator model (Remote work)	NIE through remote work	NDE of income quintile	Outcome model (well-being)	Mediator model (Remote work)	NIE through remote work	NDE of income quintile	Outcome model (well-being)	Mediator model (Remote work)	NIE through remote work	NDE of income quintile
	[1]	[2]	[3]	[4]	[5]	[6]	[7]	[8]	[9]	[10]	[11]	[12]	[13]	[14]	[15]	[16]
Cross-term of pre-pandemic income quintile and year 2022 dummy																
III * Y2022	0.0558	0.0729***	− 0.0464***	0.0558	− 0.0892	0.0725***	0.0206***	− 0.0892	− 0.0400	0.0725***	0.0200**	− 0.0400	− 0.121	0.100***	0.0262**	− 0.121
	(0.202)	(0.0100)	(0.0177)	(0.202)	(0.0836)	(0.01000)	(0.00758)	(0.0836)	(0.0941)	(0.01000)	(0.00902)	(0.0941)	(0.137)	(0.0138)	(0.0129)	(0.137)
IV * Y2022	− 0.204	0.123***	− 0.0782***	− 0.204	0.00148	0.123***	0.0350***	0.00148	0.151*	0.122***	0.0337**	0.151*	0.0577	0.151***	0.0393**	0.0577
	(0.192)	(0.0114)	(0.0289)	(0.192)	(0.0770)	(0.0114)	(0.0122)	(0.0770)	(0.0879)	(0.0114)	(0.0143)	(0.0879)	(0.123)	(0.0139)	(0.0189)	(0.123)
V * Y2022	− 0.257	0.164***	− 0.104***	− 0.257	0.126	0.165***	0.0469***	0.126	0.172*	0.165***	0.0454**	0.172*	− 0.00646	0.200***	0.0523**	− 0.00646
	(0.190)	(0.0139)	(0.0383)	(0.190)	(0.0779)	(0.0139)	(0.0165)	(0.0779)	(0.0889)	(0.0139)	(0.0199)	(0.0889)	(0.125)	(0.0165)	(0.0255)	(0.125)
Remote work dummy	− 0.637***				0.285***				0.276**				0.261**			
	(0.227)				(0.0968)				(0.118)				(0.124)			
Observations	8,314				8,343				8,347				5,781			

1) ***, **, and * indicate statistical significance at the 1%, 5%, and 10% levels, respectively

2) Age, age squared, sex, educational level, pre-pandemic income quintile, and Year 2022 dummy are controlled

3) The sample is restricted to workers only for columns [13]–[16]

*Source:* Authors’ calculation using the JHPS

## Footnotes

1 Regarding the immediate impact on well-being inequality, Delhey et al. (2023) find that the COVID-19 pandemic did not increase inequality in life satisfaction in Germany and the United Kingdom from 2020 to 2021. However, Sudo (2022) reports that inequality in life satisfaction increased during the COVID-19 pandemic in Japan in 2020.

2 Several studies have investigated the positive associations between wage and IT skill or abstract tasks. For example, Hanushek et al. (2015) and Falck et al. (2021) find that IT skills significantly increase wages, even after addressing causality. Acemoglu and Autor (2011) and Kobayashi and Yamamoto (2020) find a positive association between abstract tasks and wage at the individual worker’s level.

3 The JHPS questionnaire includes several questionnaire items on household income: household gross income, household disposable income, and amount of household income by each income source. The first two questions directly ask for the total amount of household income, whereas in the other questions, the total amount is obtained by adding each source of income. We mainly use household gross income. In the JHPS2022, after data cleaning, the correlation coefficient of household gross and disposable incomes is 0.96, and that of household gross income and total amount of each source of income is 0.95.

4 In Fig. 2, the violin plot is a mixture of box and kernel density plots, and it shows the distribution, mode (peak of the distribution), and median (white dot) in the data.

5 Since there is no golden rule for measuring well-being inequality, the other indices for well-being inequality, such as Jenkins’ inequality index (Jenkins, 2020) and percent maximum standard deviation (Delhey & Kohler, 2011), are also calculated. The results are shown in the Appendix. The trends are similar among those indices.

6 The regression analysis of job satisfaction restricts the sample to only workers.

7 In causal mediation analysis, we used the “mediate” command in STATA18 [Software].

## Appendix

See Table 6.

**Table 6:** Other well-being inequality indices

	2019	2020	2021	2022
*K6*				
Jenkins’ inequality index (upward-looking status)	0.615	0.612	0.623	0.622
Percent maximum standard deviation	0.496	0.502	0.512	0.519
Standard deviation	4.587	4.622	4.970	5.052
Coefficient of variation	4.833	4.975	5.004	5.130
*Health satisfaction*				
Jenkins’ inequality index (upward-looking status)	0.596	0.596	0.597	0.594
Percent maximum standard deviation	0.477	0.480	0.467	0.457
Standard deviation	2.361	2.364	2.311	2.259
Coefficient of variation	0.970	0.958	0.931	0.890
*Life satisfaction*				
Jenkins’ inequality index (upward-looking status)	0.586	0.587	0.587	0.585
Percent maximum standard deviation	0.427	0.434	0.427	0.424
Standard deviation	2.072	2.105	2.078	2.064
Coefficient of variation	0.693	0.715	0.703	0.695
*Job satisfaction (workers only)*				
Jenkins’ inequality index (upward-looking status)	0.597	0.598	0.597	0.596
Percent maximum standard deviation	0.480	0.475	0.472	0.475
Standard deviation	2.392	2.359	2.346	2.364
Coefficient of variation	1.047	1.005	0.993	1.024

*Source:* Authors’ calculation using the JHPS
